# Cognitive Impairment and Self-Reported Dementia in UK Retired Professional Soccer Players: A Cross Sectional Comparative Study

**DOI:** 10.1186/s40798-023-00588-2

**Published:** 2023-06-08

**Authors:** Tara-Mei Povall Macnab, Shima Espahbodi, Eef Hogervorst, Ahmed Thanoon, Gwen Sascha Fernandes, Bonnie Millar, Ashley Duncan, Maria Goodwin, Mark Batt, Colin W. Fuller, Gordon Fuller, Eamonn Ferguson, Tobias Bast, Michael Doherty, Weiya Zhang

**Affiliations:** 1 Academic Rheumatology, School of Medicine, Clinical Sciences Building, University of Nottingham, Nottingham City Hospital, Hucknall Road, Nottingham, NG5 1PB UK; 2 Versus Arthritis Centre for Sport, Exercise and Osteoarthritis, University of Nottingham, Nottingham, UK; 3 National Centre for Sport and Exercise Medicine (NCSEM), School of Sport, Exercise and Health Sciences, Loughborough University, Loughborough, UK; 4 Pain Centre Versus Arthritis, Academic Rheumatology, City Hospital, Nottingham, UK; 5 Population Health Science, Bristol Medical School, University of Bristol, Bristol, UK; 6 Colin Fuller Consultancy Ltd, Sutton Bonington, UK; 7 Centre for Urgent and Emergency Research, University of Sheffield, Sheffield, UK; 8 School of Psychology, University of Nottingham, Nottingham, UK; 9 Neuroscience@Nottingham, University of Nottingham, Nottingham, UK; 10 NIHR Nottingham Biomedical Research Centre, University of Nottingham, Nottingham, UK

**Keywords:** Soccer players, General population, Cognitive function, Self-reported dementia

## Abstract

**Background:**

Previous studies based on death certificates have found professional soccer players were more likely to die with neurodegenerative diseases, including dementia. Therefore, this study aimed to investigate whether retired professional male soccer players would perform worse on cognitive tests and be more likely to self-report dementia diagnosis than general population control men.

**Methods:**

A cross-sectional comparative study was conducted between August 2020 and October 2021 in the United Kingdom (UK). Professional soccer players were recruited through different soccer clubs in England, and general population control men were recruited from the East Midlands in the UK. We obtained self-reported postal questionnaire data on dementia and other neurodegenerative diseases, comorbidities and risk factors from 468 soccer players and 619 general population controls. Of these, 326 soccer players and 395 general population controls underwent telephone assessment for cognitive function.

**Results:**

Retired soccer players were approximately twice as likely to score below established dementia screening cut-off scores on the Hopkins Verbal Learning Test (OR 2.06, 95%CI 1.11–3.83) and Verbal Fluency (OR 1.78, 95% CI 1.18–2.68), but not the Test Your Memory, modified Telephone Interview for Cognitive Status, and Instrumental Activities of Daily Living. Analyses were adjusted for age, education, hearing loss, body mass index, stroke, circulatory problems in the legs and concussion. While retired soccer players were younger, had fewer cardiovascular diseases and other morbidities and reported healthier lifestyles, 2.8% of retired soccer players reported medically diagnosed dementia and other neurodegenerative disease compared to 0.9% of controls (OR = 3.46, 95% CI 1.25–9.63) after adjustment for age and possible confounders.

**Conclusions:**

UK male retired soccer players had a higher risk of performing below established cut-off scores of dementia screening tests and were more likely to self-report medically diagnosed dementia and neurodegenerative diseases, despite having better overall physical health and fewer dementia risk factors. Further study is needed to determine specific soccer-related risk factors.

## Key Points

- Retired UK professional male soccer players had poorer cognitive performance than general population control men.
- Retired UK professional soccer players reported more dementia than general population control men.
- Further investigation is needed to identify specific risk factors associated with the increased risk in soccer players.

## Background

Playing contact sports, including amateur and professional soccer, has been associated with brain damage and neurodegenerative diseases, such as dementia and Parkinson's disease [1–6]. Repetitive mild traumatic brain injury (mTBI) is suggested to promote chronic traumatic encephalopathy (CTE) and other dementia pathology in sports [7–9]. A systematic review and meta-analysis found that TBI almost doubled the risk of dementia [10]. However, in most of these studies, soccer players were not well represented. A further systematic review with meta-analysis concluded that athletes in contact sports might have a higher risk of mortality from neurodegenerative disease than the general population or athletes in non-contact sports [11]. The UK-based Football's InfluencE on Lifelong health and Dementia risk (FIELD) study [12] examined medical health records of 7676 Scottish former professional soccer players and 23,028 matched controls from the general population. The study found that retired professional soccer players had a 3.5-fold increased risk of neurodegenerative disease reported on their death certificate and were more likely to have been prescribed dementia-related medication in their lifetime [13, 14]. The results from the FIELD study were supported by another large data study in France [15], where French soccer players’ standard mortality rate (SMR) from unspecified dementia and Alzheimer’s disease was 3.38-fold higher than that of national population controls.

Due to increased awareness and media interest, retired soccer players may be more likely to actively seek a medically confirmed dementia diagnosis [16]. Conversely, it could be posited that retired soccer players may be deterred from completing a questionnaire focused solely on dementia and cognitive impairment. Therefore, we developed a general questionnaire enquiring about self-reported morbidity, including neurodegenerative and cardiovascular diseases and osteoarthritis, to address such potential selection bias. Furthermore, we used objective cognitive-based dementia screening tests in older retired soccer players and general population controls to reduce recall bias. Cardiovascular disease and its risk factors, including older age, low education, hypertension, obesity, diabetes and depression, are associated with Alzheimer's disease, the most common type of dementia [10, 17], and were, therefore, incorporated in our study. We hypothesized that retired professional male soccer players would perform worse on dementia screening cognitive tests than general population control men independent of these established risk factors and would self-report a higher prevalence of neurodegenerative disease, including dementia.

## Methods

We have fully described elsewhere the protocol for the Foot/ankle Osteoarthritis and Cognitive impairment in UK Soccer players (FOCUS) study [18]. The East Midlands-Leicester Central Research Ethics Committee approved this cross-sectional comparative observation study (REC Ref: 19/EM/0354). Informed consent was obtained from participants before the study onset. Participants were recruited from the previous study population [19], in which retired male professional soccer players living throughout the UK and general population control men from the East Midlands were compared for the prevalence of knee osteoarthritis. The sample size was estimated according to a 6.4% prevalence of neurodegenerative disease in the general population [20] and the 3.5 times increased risk in soccer players from the recent FIELD study [12], whereby 250 per group would give 90% power with 5% type I error for this study [18].

Of 1207 male soccer player participants in our previous study [19], 878 indicated a willingness to be approached for further research. Of 1443 male control participants who attended the third-year follow-up of the previous prospective cohort study in the general population, 1060 indicated willingness to participate in further research. These participants were sent the FOCUS postal questionnaire between August 2020 and May 2021. Participants who completed the questionnaire were subsequently invited to a telephone-based assessment of their cognitive function, irrespective of any cognitive symptoms they might have (these assessments were completed by October 2021). Due to the impact of the COVID-19 pandemic during the study timeframe, face-to-face assessments were not possible, so the original FOCUS protocol was adapted to allow for clinical assessment by telephone for all participants [18].

The FOCUS postal survey included questions on general health and self-reported physician-diagnosed long-term conditions, including cardiovascular and neurodegenerative disease (which was a composite of Alzheimer's disease, Parkinson's disease and other types of dementia) [16] and the Test Your Memory (TYM) [21] instrument to detect possible cognitive impairment in participants through self-report. The TYM was developed and validated to allow self-report assessment of memory dysfunction, which is common in dementia. Potential confounding variables for the association between screened dementia and playing professional soccer included self-reported age (in years), height and weight for body mass index (BMI), smoking history (never, past and current), alcohol use (2–3 units/day vs > 2–3 units/day according to the UK guidelines [22]), concussion (yes/no), head injury (yes/no), comorbidities (self-reported medically diagnosed long-term conditions yes/no), and socioeconomic status (SES) by postcode [23]. *Concussion* was defined as “a direct or indirect blow to the head, face, or neck causing impact to the brain, followed by a variety of symptoms that may include any of the following: headache, dizziness, loss of balance, blurred vision, “seeing stars”, feeling in a fog or slowed down, memory problems, poor concentration, and nausea or throwing up”. *Head injury* was defined as a serious head injury/impact that needed medical care/hospitalization. In addition, the survey included questions about general health, which, in part, was aimed at increasing the response rate and minimizing the impact of attention bias created by a single disease survey focused on neurodegenerative disease [18].

A cognitive screening assessment by telephone was administered to participants who indicated in their returned questionnaire their willingness to participate in this assessment. The screening included the modified Telephone Interview for Cognitive Status (TICS-M) [24, 25], Hopkins Verbal Learning Test (HVLT) [26, 27], Verbal Fluency Test (VFT) [28–30], and all eight areas of functioning of the Instrumental Activities of Daily Living (IADL) questionnaire [31]. The assessment also determined the participant’s level of education. The TICS-M was originally developed and validated for dementia screening by telephone [26]. A cut-off of 21 was suggested for optimal sensitivity and specificity for dementia [27]. The HVLT was initially developed for face-to-face auditory presentation of stimuli, with a cut-off for 15 established as the most optimal for dementia screening [26, 32, 33]. The HVLT was considered appropriate for use by telephone [34], as was the VFT (cut-off of 20) [35] and IADL (cut-off of 16) [36]. We used the immediate total recall score (total of 3 trials of 12 words recalled) of the HVLT as this showed equal validity in dementia screening to the delayed recall score [26], and this saved time. Hearing ability was tested by self-reporting (Do you have hearing loss?) and asking participants to repeat the sentence ‘No ifs buts or maybes’. If the last s was not pronounced or other words presented auditorily were not repeated in the tests, hearing loss was defined and adjusted for in the analysis as a potential confounder. To reduce inter-tester variability, testers were trained by one of the authors (EH), who has > 30 years’ experience in neuropsychological screening for dementia, to further ensure the reliability and validity of the tests. There were no systematic differences in cognitive test scores between testers.

### Statistical Analysis

To compare retired soccer players with controls, Chi-Square tests were employed for categorical data, and independent t-tests or Mann–Whitney U- tests for continuous data depending on the normality tests. Receiver Operating Curve (ROC) analyses were used to investigate whether self-reported dementia diagnoses were associated with previously established (see above) reference cut-off scores for dementia screening on the cognitive tests in this population. Logistic regression was used to calculate the odds ratio (OR) and 95% confidence interval (CI), where cognitive impairment, according to the reference cut-offs, was the dependent variable, whereas being a soccer player and other risk factors were independent variables. A stepwise forward approach was used to fit the model where being a soccer player or control was entered in step 1, age and education were entered in step 2, and potential confounding variables, which were significantly different between retired soccer players and controls. were entered stepwise forward in step 3. Hearing loss (a risk factor for dementia [37, 38]) was also entered to control for the use of telephone-based cognitive screening tests, as this could have affected performance. We also investigated self-reported neurodegenerative disease (Alzheimer’s or Parkinson’s disease and other types of dementia) using similar logistic regression analyses. Analyses were performed using SPSS 28.0 with a *p*-value of 0.05, indicating significance. We followed the Strengthening the Reporting of Observational Studies in Epidemiology (STROBE) to report this observational study [39].

## Results

**Fig. 1:**
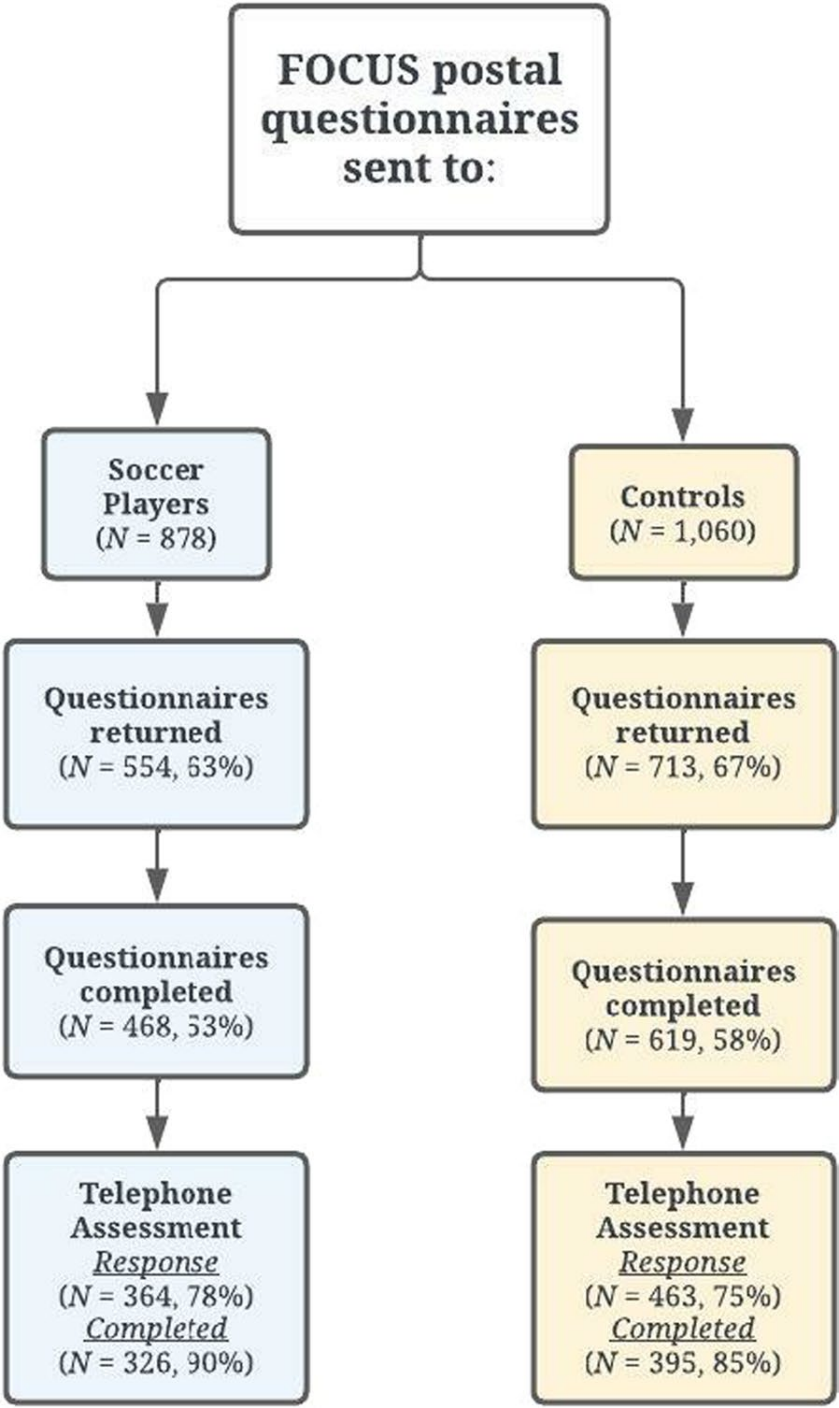
Recruitment of retired professional soccer players and general population controls

Of 878 soccer players available for this study (source population), 468 completed questionnaires and 326 completed telephone assessments for cognitive function. Of 1060 general population controls available for this study, 619 completed questionnaires and 395 completed telephone assessments for cognitive function (Fig. 1). There were no differences in age, BMI and socioeconomic status (SES) among the source population, questionnaire sample and cognitive test sample in soccer players (“Appendix A”). Similarly, no differences were observed in controls for age and BMI between the source population, questionnaire sample and cognitive test sample. However, a difference in SES was observed between the source population and the cognitive test sample, but not in other pair comparisons in controls (“Appendix B”).

Table 1 shows the participant characteristics and key self-reported survey data. Retired soccer players were, on average, four years younger than controls, had significantly higher socioeconomic status (SES), and had higher mean BMI. In addition, retired soccer players more frequently reported lower limb peripheral vascular disease and stroke but not cerebrovascular diseases, such as transient ischemic attack (TIA). Cardiovascular comorbidities and other risk factors for dementia were found to occur more frequently in controls, including diabetes mellitus, hypertension, high cholesterol levels, myocardial infarction, depression, hearing loss and current smoking. There were no differences in alcohol use between retired soccer players and controls. Retired soccer players were significantly more likely to have experienced concussions and head injuries than controls (Table 1). Of neurodegenerative diseases, self-reported physician-diagnosed dementia and Alzheimer's disease were significantly more common in retired soccer players than controls, but only controls reported a few diagnoses of Parkinson's disease. In addition, TYM scores were lower in retired soccer players, suggesting they were more likely to self-report memory problems.

**Table 1:** Characteristics of ex-professional soccer players and general population (control group) participants

	Soccer players (*N* = 468)	General Population (*N* = 619*)*	*P* value
Age (years), mean (SD)	63.7 (10.5)	68.4 (9.3)	**< .001**^**c**^
Socioeconomic status, mean (SD)	7.5 (2.4)	6.6 (3.0)	**< .001**^**c**^
BMI > 25, n/total (%)	341/459 (74.3)	373/585 (63.8)	**< .001**^**b**^
Stroke, n (%)	19 (4.1)	14 (2.3)	.09^b^
Transient Ischaemic Attack, n (%)	13 (2.8)	15 (2.4)	.72^b^
Head injury, n/total n (%)	218/456 (47.8)	142/610 (23.3)	**< .001**^**b**^
Concussion, n (%)	247/465 (53.1)	97/611 (15.9)	**< .001**^**b**^
Hearing loss, n (%)	48 (10.3)	117 (18.9)	**< .001**^**b**^
Diabetes mellitus, n (%)	14 (3.0)	67 (10.8)	**< .001**^**b**^
Hypertension, n (%)	107 (22.9)	217 (35.1)	**< .001**^**b**^
Myocardial infarction, n/total n (%)	14 (3)	35 (5.7)	**< .04**^**b**^
Circulatory problems in legs, n (%)	50 (10.7)	39 (6.3)	**< .01**^**b**^
High cholesterol, n (%)	84 (17.9)	147 (23.7)	**.02**^**b**^
Current smokers, n/total n (%)	12/456 (2.6)	32/606 (5.3)	**< .001**^**b**^
Drinks > 2–3/day, n (%)	31/146 (21.2)	58/255 (22.7)	.73^b^
Depression, n (%)	35 (7.5)	71 (11.5)	**.03**^**b**^
Dementia/Alzheimer’s, n(%)	13 (2.8)	2 (0.3)	**< .001**^**b**^
Parkinson’s, n (%)	0	4 (0.6)	.08^b^
Neurodegenerative disease, n (%)	13 (2.8)	6 (0.9)	**.02**^**b**^
TYM, mean (SD)	37.4 (4.2)	38.9 (3.0)	**< .001**^**c**^

*p* ≤ 0.05 is defined as significant and marked in bold

*BMI* body mass index, *TYM* test your memory

^*^Two-sided testing

^a^Independent samples T-test

^b^Chi Square test

^c^Mann-Whitney U test

Mean performance scores of TICS-M and HVLT did not differ significantly between retired soccer players and controls (Table 2). However, the mean performance scores of VFT and IADL were lower in retired soccer players. In addition, education but not SES was lower in retired soccer players.

**Table 2:** Telephone interview and cognitive tests

	Soccer players (*N* = *326*)	General population (*N* = *395*)	*P* value
Hearing affected n (%)	73 (29.4)	106 (26.7)	.57^a^
Level of Education (Md, IQR)	3 (3)	6 (3)	< .001^b^
Cognitive tests			
TICS-M, mean (SD)	26.2 (4.7)	26.3 (4.1)	.57^b^
VFT, mean (SD)	21.4 (5.8)	24.7 (6.7)	< .001^b^
HVLT, mean (SD)	22.4 (6.0)	22.1 (5.6)	.53^b^
IADL, mean (SD)	15.2 (2.1)	15.5 (1.2)	.54^b+c^

Level of education 3 = Secondary school (GCSE level), while 6 indicates Diploma level

*Md* Median, *IQR* Interquartile Range, *SD* Standard Deviation, *TICS-M* the modified telephone interview for cognitive status, *HVLT* Hopkins verbal learning test, *IADL* instrumental activities of daily living, *VFT* verbal fluency test

^a^Chi Square Test

^b^Independent Samples T-test

^c^Mann-Whitney U Test

**Fig. 2:**
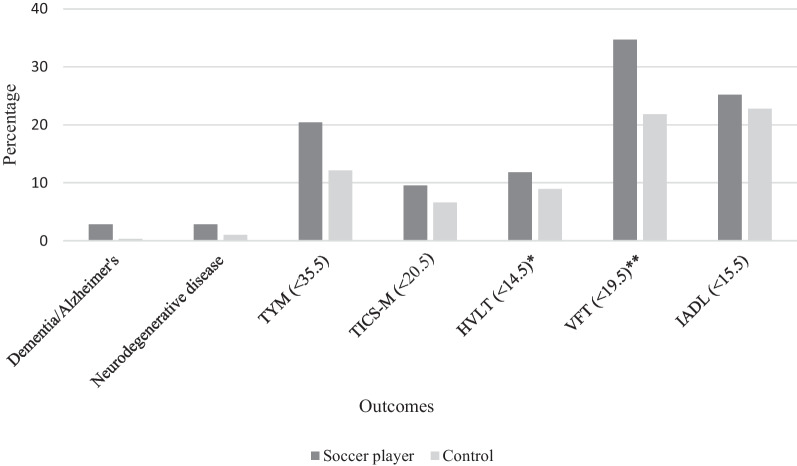
Percentages of retired soccer players versus controls who self-reported a medical diagnosis of dementia/Alzheimer’s, neurodegenerative disease or cognitive impairment based on established cut-offs of cognitive tests.*P ≤ .05; **P ≤ .001

ROC analyses demonstrated significant Area Under the Curves (AUC) for all cognitive tests against self-reported dementia (*p* < 0.001), with an optimal cut-off score of 20.5 (AUC = 0.90) for TICS-M, 14.5 (AUC = 0.98) for HVLT, 19.5 (AUC = 0.89) for VFT, 15.4 (AUC = 0.94) for IADL and 35.5 (AUC = 0.92) for TYM, respectively. These were all similar or identical to the previously established cut-off scores for these dementia screening cognitive tests [26, 27, 32, 33, 35, 36]. Figure 2 demonstrates the prevalence of self-reported dementia/Alzheimer’s, neurodegenerative disease and the cognitive test performance below the cut-offs.

Using the established dementia screening test cut-off scores as an outcome measure in logistic regression analyses, an HVLT total recall score below that score indicative of dementia was found to be more likely in retired soccer players, independent of age, education and other potential confounding factors. Apart from age, no other variables were associated with low HVLT scores in this model. Performing below the VFT cut-off score was also associated with playing professional soccer in the past, independent of age and education, as well as other potential confounding factors, such as having a higher BMI, having experienced a stroke, and reporting hearing loss, which all had independent contributions to dementia risk in this model (Table 3). Scoring below the IADL cut-off was associated with older age, having lower obtained education and circulatory problems in the legs, but not with having been a professional soccer player (Table 3).

**Table 3:** Odds ratio of self-reported neurodegenerative disease diagnosis and cognitive impairment between soccer players and controls

Outcomes	Odds ratio (95% confidence interval)		
	Crude	Model 1	Model 2
Neurodegenerative disease	2.92 (1.10, 7.74)	4.41 (1.63, 11.91)	3.46 (1.25, 9.63)
TYM (< 35.5)	1.87 (1.34, 2.60)	1.51 (0.95, 2.38)	1.55 (0.97, 2.46)
TICS-M (< 20.5)	1.49 (0.87, 2.57)	1.37 (0.73, 2.57)	1.41 (0.73, 2.73)
HVLT (< 14.5)	1.37 (0.84, 2.24)	1.64 (0.92, 2.93)	2.06 (1.11, 3.83)
VFT (< 19.5)	1.91 (1.37, 2.65)	1.84 (1.24, 2.72)	1.78 (1.18, 2.68)
IADL (< 15.5)	1.14 (0.81, 1.61)	1.42 (0.94, 2.14)	1.24 (0.81, 1.89)

Model 1. Adjusted by age and education

Model 2. Adjusted by age, education, Body Mass Index (BMI) cut-off of < 25, stroke, concussion, circulation problems, hearing loss (only for telephone assessment)

*TYM* test your memory, *TICS-M* the modified telephone interview for cognitive status, *HVLT* Hopkins verbal learning test, *VFT* Verbal fluency test, *IADL* instrumental activities of daily living

Logistic regression analyses were also performed to predict medically diagnosed neurodegenerative disease risk in retired soccer players with adjustments for age and education in Model 1 and adjustments for other potential confounding factors reported to be higher in retired soccer players than controls (higher BMI, having experienced a concussion, having circulatory problems, and having a stroke) in Model 2. Education was removed from the final analyses as it was non-significant and resulted in a reduced number of respondents because it was only included during cognitive assessments by phone in the sub-group tested (Table 3). The results demonstrated that being a retired soccer player remained in the model as a significant and independent risk factor for neurodegenerative disease (OR = 3.46, 95% CI = 1.25–9.63, *p* = 0.02). Age also contributed independently to the risk for neurodegenerative disease (OR = 1.18, 95% CI = 1.10–1.27, *p* < 0.001), but none of the other variables were entered in the model. Dementia risk was not analyzed separately- because there were only two self-reported dementia cases in controls- to avoid overestimation of the relative risk of dementia.

## Discussion

This study compared objective cognitive impairment measurements indicative of dementia between retired male professional soccer players and general population men. The key findings are that retired soccer players were more likely to perform below dementia cut-off scores on the more complex HVLT and VFT screening tests. In addition, the prevalence of self-reported neurodegenerative disease, including dementia/Alzheimer's disease diagnosis, was higher in retired soccer players than in controls. These results were independent of the major risk factors for dementia, including age, education, and comorbidities.

Our results for the self-reported diagnosis of neurodegenerative disease are similar to those reported in Morales and colleagues’ systematic review with meta-analyses [11] and the FIELD study of retired Scottish soccer players [7, 12] with a 3.5 increased risk for neurodegenerative disease, including Alzheimer's, dementia. However, no cases of Parkinson's disease were reported among retired soccer players and only 4 cases reported in controls, that made a relevant comparison impossible for this subset. In addition, no cases of motor neuron disease were reported in both players and controls. Further study on this subset is still needed.

The FIELD study [12], which used community health index number registry data matched to soccer player’s databases, also found that Scottish professional soccer players died earlier of neurodegenerative diseases compared with controls after controlling for age, player position, morbidity (e.g., stroke) and SES index using the Scottish index of multiple deprivations combining education, postcode level data on income, health, housing access to local amenities and crime). We collected SES data through postcodes [23] for an index of multiple deprivation scores and education separately at the individual level. We found that SES was higher, but education was lower in retired professional soccer players than in the general population control men. Worse socioeconomic deprivation is associated with poorer cognition, while higher education is associated with better cognition [40]. When we included these two variables in the model separately for adjustment, we found that education remained significant (and was negatively associated with the cognitive outcomes) along with age in the model, whereas SES became insignificant when age was included. Therefore, unlike the FIELD study, we used individuals’ education, rather than SES, as a potential confounding factor for adjustment. Despite the fact that retired soccer players were, on average, five years younger than controls, performance on some dementia screening tests remained significantly lower when controlling for age and education. Cognitive screening tests employed via telephone assessment in this study showed similar cut-off scores to those used when face-to-face testing in other validation studies [26, 27, 32, 33, 35, 36], further supporting the validity of our dementia screening test results. In the present study, of these tests, only the more complex HVLT and VFT were more commonly scored significantly below the dementia impairment cut-off in retired soccer players. The HVLT and VFT involve verbal recall and executive functions, which could be hypothesized to be affected more strongly by repeated mTBI or CTE experienced by heading the ball [41–43]. The other dementia screening instruments, with more simple memory tests and questionnaires sensitive to dementia (TICS-M, IADL, TYM), were not different between retired soccer players and controls in controlled analyses adjusting for confounds, which requires further investigation.

Notably, the overall health of retired older soccer players was much better than that of controls in our study. This was in line with the FIELD study [12] and a more recent study in France [15], where all-cause mortality was lower in professional soccer players than in age-matched controls. This may reflect the healthier lifestyle and good fitness levels sustained post-retirement by the retired soccer players, who were less likely to have other long-term conditions, such as cardiovascular diseases, diabetes and cancer. Given the overall good health profile of the retired professional soccer players, the increased risk of cognitive impairment and dementia becomes more prominent and specific. Further studies on soccer-specific risk factors are needed.

There are several limitations to this study. Firstly, this is only a cross-sectional study which cannot establish a causal association between playing soccer and dementia. Although we had controlled for common measurable confounding bias, a further cohort study is still required. Secondly, not all available participants underwent the telephone-based cognitive tests. This brings into question the representativeness of the samples from both soccer players and controls. Therefore we compared both in soccer players and controls, the available source population, the questionnaire sample and the cognitive test sample (“Appendix A, Appendix B”). There were no differences in age, BMI and SES among the three groups of soccer players, and only a slight difference was observed for SES in controls, specifically between the source population and cognitive test sample. This suggests that both the cognitive tests and self-reported outcomes are generally representative. Thirdly, like many other retrospective self-reported studies, potential recall bias cannot be ruled out. We therefore collected more objective measures-cognitive performance tests—to minimize this bias. Fourthly, self-reported dementia is prone to misclassification bias. However, we undertook a validation analysis against the cognitive performance tests. Similar cut-offs were obtained as recommended in other face-to-face validation studies, suggesting that self-reported dementia was less likely to be biased due to recall issues in those with dementia. However, with the low average age of this sample, there was a very low number of people who had self-reported physician-diagnosed dementia in both groups, with 97% of retired soccer players and 99% of controls not reporting dementia. The overall self-reported dementia prevalence in controls was lower for this age group than expected based on Office of National Statistics (ONS) data [43]. People with established dementia may be less likely, or unable to respond to the postal questionnaire, which could result in a lower prevalence of dementia in our responders. Such left censorship bias would be expected to affect both groups. Therefore, the prevalence of self-reported dementia may be underestimated, and caution must be taken when interpreting this outcome. Lastly, as mentioned it could be the case that because of increased media attention for this topic, professional football players with cognitive impairments would be more willing to enroll in the study as they might be interested and/or worried more. However, the post-mortem confirmed data for dementia from earlier studies are in line with our increased risk outcomes, which suggests that this is a less likely reason for our findings.

## Conclusion

In conclusion, this study found that retired UK male professional soccer players were more likely to have self-reported neurodegenerative disease, specifically dementia, than general population control men. This result was adjusted for confounding factors and was supported by objectively measured cognitive function screening tests. Further study is recommended to confirm these results and to identify the specific risk factors related to the increased risk of dementia in professional soccer players.

## Data Availability

Data for Foot/ankle Osteoarthritis and Cognitive impairment in UK Soccer players (FOCUS) is available upon request and subject to the data transfer agreement (DTA) between the University of Nottingham and data requester.

## Appendix A

See Table 4.

**Table 4:** Comparison among source population, questionnaire and cognitive test samples in soccer players

	Source population	Questionnaire sample	Cognitive sample	*P*-value*
No. participants	878	468	326	–
Age (years), mean (SD)	63.40 (11.35)	63.71 (10.47)	63.33 (10.00)	.86
BMI (kg/m2), mean (SD)	27.34 (3.00)	27.22 (3.00)	27.12 (2.88)	.49
SES, mean (SD)	7.41 (2.42)	7.50 (2.40)	7.62 (2.36)	.45

*BMI* body mass index, *SES* Socioeconomic Status, measured according to postcode

*AVOVA test

## Appendix B

See Table 5.

**Table 5:** Comparison among source population, questionnaire and cognitive test samples in general population controls

	Source Population	Questionnaires completed	Cognition tested	*P*-value*
No. participants	1060	619	395	–
Age (years), mean (SD)	68.78 (9.53)	68.07 (10.27)	68.30 (8.91)	.32
BMI (kg/m2), mean (SD)	27.19 (4.27)	26.80 (4.35)	26.94 (4.36)	.19
SES, mean (SD)	6.31 (3.02)	6.59 (2.98)	6.81 (2.94)	.02^#^

*BMI* body mass index, *SES* Socioeconomic Status, measured according to postcode

*AVOVA test

^#^post-hoc paired test showed that the difference was only observed between source population and cognition tested group

## Abbreviations

UK: United Kingdom
FOCUS: Foot/ankle Osteoarthritis and Cognitive impairment in the UK Soccer players
HVLT: Hopkins Verbal Learning Test
VFT: Verbal Fluency Test
TYM: Test Your Memory
TICS-M: Modified Telephone Interview for Cognitive Status
LDAL: Instrumental Activities of Daily Living
BMI: Body Mass Index
SES: Socioeconomic Status
ROC: Receiver Operating Curve
AUC: Area Under the Curves
OR: Odds Ratio
CI: Confidence Interval
SD: Standard Deviation
Md: Median
IQR: Interquartile Range
STROBE: Strengthening the Reporting of Observational Studies in Epidemiology

## References

[1] Neal J, Hutchings PB, Phelps C, Williams D (2022). Football and Dementia: understanding the link. Front Psychiatry.

[2] Matser JT, Kessels AGH, Lezak MD, Troost J (2001). A dose-response relation of headers and concussions with cognitive impairment in professional soccer players. J Clin Exp Neuropsychol.

[3] Matser EJT, Kessels AG, Lezak MD, Jordan BD, Troost J (1999). Neuropsychological impairment in amateur soccer players. JAMA.

[4] Tysvaer AT, Løchen EA (1991). Soccer injuries to the brain: a neuropsychologic study of former soccer players. Am J Sports Med.

[5] Tysvaer AT, Storli O-V, Bachen NI (1989). Soccer injuries to the brain. A neurologic and electroencephalographic study of former players. Acta Neurol Scand.

[6] Sortland O, Tysvaer AT (1989). Brain damage in former association football players. Neuroradiology.

[7] Baugh CM, Stamm JM, Riley DO, Gavett BE, Shenton ME, Lin A (2012). Chronic traumatic encephalopathy: neurodegeneration following repetitive concussive and subconcussive brain trauma. Brain Imaging Behav.

[8] Gavett BE, Stern RA, McKee AC (2011). Chronic traumatic encephalopathy: a potential late effect of sport-related concussive and subconcussive head trauma. Clin Sports Med.

[9] McKee AC, Cantu RC, Nowinski CJ, Hedley-Whyte ET, Gavett BE, Budson AE (2009). Chronic traumatic encephalopathy in athletes: progressive tauopathy after repetitive head injury. J Neuropathol Exp Neurol.

[10] Livingston G, Huntley J, Sommerlad A, Ames D, Ballard C, Banerjee S (2020). Dementia prevention, intervention, and care: 2020 report of the Lancet Commission. Lancet (London, England).

[11] Morales JS, Valenzuela PL, Saco-Ledo G, Castillo-Garcia A, Carabias CS, Paul M (2022). Mortality risk neurodegenerative disease in sports associated with repetitive head impacts: preliminary findings from a systematic review and meta-analysis. Sp Med.

[12] Mackay DF, Russell ER, Stewart K, MacLean JA, Pell JP, Stewart W (2019). Neurodegenerative disease mortality among former professional soccer players. N Engl J Med.

[13] Russell ER, Mackay DF, Stewart K, MacLean JA, Pell JP, Stewart W (2021). Association of field position and career length with risk of neurodegenerative disease in male former professional soccer players. JAMA Neurol.

[14] Iacobucci G (2021). Dementia risk in professional footballers is linked to player position and career length, study finds. BMJ.

[15] Orhant E, Carling C, Chapellier JF, Marchand JL, Pradat PF, Elbaz A (2022). A retrospective analysis of all-cause and cause-specific mortality rates in French male professional footballers. Scand J Med Sci Sp.

[16] Cunningham J, Broglio SP, O'Grady M, Wilson F (2020). History of sport-related concussion and long-term clinical cognitive health outcomes in retired athletes: a systematic review. J Athl Train.

[17] Solomon A, Ngandu T, Kivipelto M, Irving K, Hogervorst E, Oliveira D, Kivipelto M (2018). From prediction to dementia prevention. New developments in dementia prevention research: state of the art and future possibilities.

[18] Espahbodi S, Fernandes G, Hogervorst E, Thanoon A, Batt M, Fuller CW (2022). Foot and ankle Osteoarthritis and Cognitive impairment in retired UK Soccer players (FOCUS): protocol for a cross-sectional comparative study with general population controls. BMJ Open.

[19] Fernandes GS, Parekh SM, Moses J, Fuller C, Scammell B, Batt ME (2018). Prevalence of knee pain, radiographic osteoarthritis and arthroplasty in retired professional footballers compared with men in the general population: a cross-sectional study. Br J Sports Med.

[20] Lobo A, Launer LJ, Fratiglioni L, Andersen K, Di Carlo A, Breteler MMB (2000). Prevalence of dementia and major subtypes in Europe: a collaborative study of population-based cohorts. Neurology.

[21] Brown J, Pengas G, Dawson K, Brown LA, Clatworthy P (2009). Self administered cognitive screening test (TYM) for detection of Alzheimer’s disease: cross sectional study. BMJ.

[22] Officers UCM. UK Chief Medical Officers’ Low Risk Drinking Guidelines

[23] 2016 [cited; Available from: https://assets.publishing.service.gov.uk/government/uploads/system/uploads/attachment_data/file/545937/UK_CMOs__report.pdf

[24] Gov.Uk. English indices of deprivation 2015. [cited; Available from: https://www.gov.uk/government/statistics/english-indices-of-deprivation-2015

[25] de Jager CA, Budge MM, Clarke R (2003). Utility of TICS-M for the assessment of cognitive function in older adults. Int J Geriatr Psychiatry.

[26] Brandt J, Spencer M, Folstein M (1988). The telephone interview for cognitive status. Neuropsychiatry Neuropsychol Behav Neurol.

[27] Hogervorst E, Combrinck M, Lapuerta P, Rue J, Swales K, Budge M (2002). The Hopkins verbal learning test and screening for dementia. Dement Geriatr Cogn Disord.

[28] Brandt J (1991). The Hopkins verbal learning test: development of a new memory test with six equivalent forms. Clin Neuropsychol.

[29] Kim N, Kim J-H, Wolters MK, MacPherson SE, Park JC (2019). Automatic scoring of semantic fluency. Front Psychol.

[30] Bolla KI, Lindgren KN, Bonaccorsy C, Bleecker ML (1990). Predictors of verbal fluency (FAS) in the healthy elderly. J Clin Psychol.

[31] Benton AL (1968). Differential behavioral effects in frontal lobe disease. Neuropsychologia.

[32] Lawton MP, Brody EM (1969). Assessment of older people: self-maintaining and instrumental activities of daily living. Gerontologist.

[33] Hogervorst E, Xin X, Rahardjo T, Shifu X (2014). The Hopkins Verbal Learning Test and detection of MCI and mild dementia: a literature review. J Alzheimer Dis Parkinson..

[34] Hogervorst E (2011). Validation of two short dementia screening tests in Indonesia.

[35] Castanho TC, Amorim L, Zihl J, Palha JA, Sousa N, Santos NC (2014). Telephone-based screening tools for mild cognitive impairment and dementia in aging studies: a review of validated instruments. Front Aging Neurosci.

[36] Rapp SR, Legault C, Espeland MA, Resnick SM, Hogan PE, Coker LH (2012). Validation of a cognitive assessment battery administered over the telephone. J Am Geriatr Soc.

[37] Mathuranath PS, George A, Cherian PJ, Mathew R, Sarma PS (2005). Instrumental activities of daily living scale for dementia screening in elderly people. Int Psychogeriatr.

[38] Tan HE, Lan NSR, Knuiman MW, Divitini ML, Swanepoel DW, Hunter M (2018). Associations between cardiovascular disease and its risk factors with hearing loss—a cross-sectional analysis. Clin Otolaryngol.

[39] Engdahl B, Aarhus L, Lie A, Tambs K (2015). Cardiovascular risk factors and hearing loss: The HUNT study. Int J Audiol.

[40] von Elm E, Altman DG, Egger M, Pocock SJ, Gøtzsche PC, Vandenbroucke JP (2007). The strengthening the reporting of observational studies in epidemiology (STROBE) statement: guidelines for reporting observational studies. The Lancet.

[41] Kouraki A, Bast T, Ferguson E, Valdes AM (2022). The association of socio-economic and psychological factors with limitations in day-to-day activity over 7 years in newly diagnosed osteoarthritis patients. Sci Rep.

[42] McCrory P, Meeuwisse W, Dvorak J, Aubry M, Bailes J, Broglio S (2017). Consensus statement on concussion in sport—the 5th international conference on concussion in sport held in Berlin, October 2016. Br J Sp Med.

[43] Gardner RC, Yaffe K (2015). Epidemiology of mild traumatic brain injury and neurodegenerative disease. Mol Cell Neurosci.

[44] Harmon KG, Drezner J, Gammons M, Guskiewicz K, Halstead M, Herring S (2013). American medical society for sports medicine position statement: concussion in sport. Clin J Sport Med.

[45] Prince M, Knapp M, Guerchet M, McCrone P, Prina M, Comas-Herrera A, et al. Dementia UK: update. 2014 [cited; Available from: https://hal.archives-ouvertes.fr/hal-03516999/document

